# Antimicrobial photodynamic therapy on *Staphylococcus aureus* and *Escherichia coli* using malachite green encapsulated mesoporous silica nanoparticles: an *in vitro* study

**DOI:** 10.7717/peerj.7454

**Published:** 2019-09-12

**Authors:** Parasuraman Paramanantham, Busi Siddhardha, Sruthil Lal SB, Alok Sharan, Abdullah A. Alyousef, Mohammed Saeed Al Dosary, Mohammed Arshad, Asad Syed

**Affiliations:** 1 Department of Microbiology, School of Life Sciences, Pondicherry University, Pondicherry, India; 2 Department of Physics, School of Physical, Chemical and Applied Sciences, Pondicherry University, Pondicherry, India; 3 Microbiology Research Group, Department of Clinical Laboratory Sciences, College of Applied Medical Sciences, King Saud University, Riyadh, Saudi Arabia; 4 Department of Botany and Microbiology, College of Science, King Saud University, Riyadh, Saudi Arabia

**Keywords:** aPDT, Biofilms, CLSM, Mesoporous silica nanoparticle, MG-MSN

## Abstract

**Background:**

Rise in the number of healthcare associated or hospital acquired infections is a major problem affecting the global healthcare sector. We evaluated superior antibacterial and antibiofilm photodynamic therapy (aPDT) using malachite green encapsulated mesoporous silica nanoparticles (MG-MSN) against *Staphylococcus aureus* and *Escherichia coli*, which are known to be major causative agents of nosocomial infections.

**Methods:**

Malachite green (MG) was encapsulated on mesoporous silica nanoparticles (MSN). Fourier-transform infrared spectroscopy, Transmission electron microscopy, and spectroscopic analysis were performed to characterize the MG-MSN. The antimicrobial efficacies of MSN, MG, and MG-MSN were investigated and the results were recorded.

**Results:**

MG-MSN was effective against both the tested bacteria. *S. aureus* was more phototoxic to MG-MSN compared to *E. coli*. The antibiofilm efficacy of MG-MSN on *E. coli* and *S. aureus* was also studied. Biofilm inhibition was 65.68 ± 2.62% in *E. coli* and 79.66 ± 3.82% in *S. aureus*. Cell viability assay, exopolysaccharides quantification, and confocal laser scanning microscopy studies also revealed the enhanced antibiofilm activity of MG-MSN when used as a potential photosensitizer for aPDT. This study can be extended to eradicate these strains from localized superficial infections and medical appliances, preventing nosocomial infections.

## Introduction

Microbial infections acquired from hospitals or healthcare centers pose a serious threat to the global healthcare sector. Increased length of hospital stay puts patients at a high risk, which in some cases can even lead to infectious diseases and is one of the reasons for the increased death rates. The Centers for Disease Control and Prevention (CDC) recognized contaminated environmental surfaces as the main sources of nosocomial infection transmission (Spagnul, Turner & Boyle, 2015). The major pathogens that cause nosocomial infections include antibiotic resistant strains of *Staphylococcus aureus*, *Escherichia coli*, and *Pseudomonas aeruginosa* (Wysocka-Król et al., 2018). These pathogens have the ability to form biofilms on biomaterials and medical devices. *S. aureus* is an opportunistic pathogen that can cause localized superficial infections to deep-seated life-threatening diseases (Mirani et al., 2013). This bacterium is linked to complications during surgical procedures such as meningitis, peritonitis, necrotizing pneumonia, osteomyelitis, bacteremia, and endocarditis. *E. coli,* a diverse and complex bacterium found in the human intestinal tract, can cause urinary tract infections, endocarditis, bacteremia, and sepsis (Ronqui et al., 2016). With the danger posed by the emergence of antibiotic resistant strains, the search for new modalities to cure bacterial infections is very much necessary. Among the various alternative treatments available, antimicrobial photodynamic therapy (aPDT) has aroused substantial interest (Kashef, Huang & Hamblin, 2017).

aPDT incorporates the interaction of light of a suitable wavelength, a light activating complex called the photosensitizer (PS), and molecular oxygen thereby eliminating the targeted cells by the *in situ* triggering of reactive oxygen species (ROS) (De Annunzio et al., 2018). ROS can be generated through either of the two mechanisms: (1) transfer of electron (type I mechanism) or (2) transfer of energy (type II mechanism) from the light-activated PS to the molecular oxygen. The ROS produced, especially the singlet oxygen reacts with almost all the cellular components and biomolecules causing cell death. The proposed mechanisms of aPDT include damage to cytoplasmic membrane, DNA, and leakage of cellular components and enzymes (Hanakova et al., 2014). aPDT is a non-toxic and non-invasive selective modality against microbial pathogens, that does not produce any photoresistant strains and also has fewer side effects compared to antibiotics (Hamblin, 2016). Various studies have revealed that, owing to the differences in their cell walls, Gram positive bacteria are more vulnerable to aPDT than Gram negative bacteria. In addition, the characteristics of PSs also influence the effect of aPDT. Cationic PSs with positive charges are more active against the negatively charged bacterial cell walls and are more efficient in antibacterial treatment (Spagnul, Turner & Boyle, 2015). There are several PSs that are known to exhibit antibacterial and antibiofilm effects; for example porphyrins, chlorins, bacteriochlorins, phenothiazines and phthalocyanines (Masiera et al., 2017). In the present study, malachite green (MG), which is a cationic photosensitizing molecule with photodynamic effects at 615 nm in the range of red light in the visible spectrum, has been used as the PS (Junqueira et al., 2010). Several studies have reported the antibacterial effect of MG in photodynamic therapy (Rosa et al., 2014; Vilela et al., 2012).

The aggregation, instability, and reduced retention time of PSs are known to negatively affect the efficiency of aPDT. In order to overcome these disadvantages, nanoparticles are used as the carriers of PSs for improving the overall effectiveness of aPDT. Among the nanovehicles, silica-based nanoparticles are very promising and of great interest to researchers. In our study, we focused on mesoporous silica nanoparticles (MSNs) as carriers of the PS (Zhou et al., 2018). MSNs are of great importance owing to their special features such as large specific surface area, tunable particle size, porosity, pore volume, biocompatibility, and high loading capacity; above all, they are safe and nontoxic in nature (Tran et al., 2018).

Silica nanoparticles have been used as nanovehicles of PSs by various researchers. Wysocka-Król et al. (2018) proved the supporting role of silica nanoparticles in aPDT for enhanced effect. The efficiency of MSNs has been proved in a study of chemo/photodynamic synergistic therapy for drug delivery and imaging by embedding carbon dots and rose bengal as PS (Liu et al., 2017). In a recent study, methylene blue-loaded MSNs have been used in the antibacterial photodynamic therapy (Planas et al., 2015).

Our present study aimed to test the effectiveness of antimicrobial photodynamic inactivation using MG encapsulated on MSNs against planktonic cells and biofilms of *S. aureus* and *E. coli.*

## Materials and Methods

### Chemicals

MSNs with a diameter of 0.5 µm and a pore size of 0.2 nm were procured from Sigma-Aldrich, Bengaluru, Karnataka, India. MG, bacterial growth essentials such as nutrient agar, Luria Bertani broth (LB broth), Luria Bertani agar (LB agar), nutrient broth, and agar-agar were procured from Hi-Media Laboratories Pvt., Ltd., Mumbai, India.

### Bacterial strains and culture conditions

The test cultures selected for the study were *S. aureus* (MCC 2408) and *E. coli* (MCC 2412). A single colony of both the test strains was inoculated into five ml of LB broth and incubated in an orbital shaking incubator at 37 °C for 24 h. The culture inoculum was adjusted using spectrophotometer reading at a wavelength of 600 nm to yield a standard bacterial suspension of 1.5 × 10^8^ colony forming units (CFU)/ml prior to each experiment.

### Encapsulation of MG on MSN

MG was encapsulated on MSNs as described previously (Perni, Martini-Gilching & Prokopovich, 2018). A solution containing MSN (25 mg) and MG (five mg) in the ratio 5:1 was prepared in 25 ml of ethanol. The resulting solution was magnetically stirred for 48 h. Then, the solution was subjected to centrifugation at 10,000 rpm for 10 min and washed thoroughly using double distilled water for three times. The collected pellet was dried at 60 °C and then stored in dark conditions after drying process.

### Characterization of MG-MSN

The absorption range of MG-MSN was compared with free MG using UV-Vis spectroscopic analysis in a wavelength range of 200–800 nm. Absorption spectrum was measured at room temperature using UV–VIS-NIR spectrophotometer (Model: varian Carry 5000). Photoluminescence spectra of MG and MG-MSN were studied by a spectrofluorometer (Jobin Yvon, Model: FLUOROLOG - FL3-11) (Gupta, Kushwaha & Chattopadhyaya, 2016). Fourier transform infrared (FTIR) spectral measurements were carried out using a FTIR spectroscope (Model: Thermo Nicolet, 6700) to identify potential functional groups present on MSN, MG, and MG-MSN at a range of 400–4,000 cm^−1^. High-resolution transmission electron microscopic (HRTEM) images were taken using an HITACHI H-8100 electron microscope (Hitachi, Tokyo, Japan). The observations were made with an accelerating voltage of 200 kV. The shape and size of MG-MSN was analyzed carefully by placing a single drop of sample suspension on a copper TEM grid coated with carbon and after evaporation of complete water moiety.

### Loading capacity (LC) of MG and entrapment efficiency (EE)

The dye loading and entrapment of MSN with MG were investigated as described previously (Yan et al., 2018). About two mg of MG-MSN was mixed in two ml of ethanol. The solution was centrifuged for 10 min at 10,000 rpm. The concentration of MG present in the supernatant solution was measured at 615 nm using UV-Vis spectrophotometer. The loading of dye and entrapment of MSN with dye was determined using the calculation given below:

\begin{eqnarray*}& & \text{Loading capacity} (\text{%})= \frac{\text{weight of loaded MG}}{\text{weight of dye encapsulated MSN}} \times 100 \end{eqnarray*}

\begin{eqnarray*}& & \text{Entrapment efficiency}= \frac{\text{weight of loaded MG}}{\text{weight of MG in feed}} \times 100. \end{eqnarray*}

### Release profile of MG

Release study was performed using an UV-Visible spectrophotometer as described previously (Planas et al., 2015). About two mg of MG-MSN was mixed with two ml of acid ethanolic media (100 mM acetic acid). The mixture was maintained under gentle shaking (100 rpm) at 37 °C. After shaking, the solution was centrifuged and MG release was determined spectroscopically at a wavelength of 615 nm. OD of the supernatant was measured at 30 min intervals with the addition of fresh medium till the supernatant became colorless. The MG release profile was measured from the standard curve of MG. The release profile of MG from MG-MSN at each interval was calculated using the formula:

\begin{eqnarray*}& & \text{Percentage of MG released}= \frac{\text{amount of dye released (mg/ml)}}{\text{amount of MG {\ndash} MSN (mg/ml)}} \times 100. \end{eqnarray*}

### Bacterial uptake study

Overnight cultures were adjusted to McFarland’s standard (1.5  × 10^8^ CFU/ml) and two mg of MG and MG-MSN each was added to two ml of bacterial culture. The experimental setup was incubated for 3 h and the samples were removed periodically every 30 min (30, 60, 90, 120, 150, and 180 min). The solution was withdrawn and centrifuged for 10 min at 10,000 rpm after every 30 min. Unbound MG and MSN were removed by washing the pellet twice with sterile PBS. Methanol (one ml) was added to the pellet and incubated at room temperature for 1 h to extract the cell bound MG. After centrifugation, the supernatant was subjected to UV-Vis spectroscopic analysis at 615 nm to measure the amount of dye bound to the cell (Usacheva et al., 2016).

The percentage of MG uptake by the bacteria was evaluated using the following equation:

\begin{eqnarray*}& & \text{Dye uptake} (\text{%})= \frac{\text{amount of MG in the dissolved pellet}}{\text{total amount of MG added}} \times 100. \end{eqnarray*}

### Photosensitizer and light source

The effects of free MG and MG-MSN on both the test cultures were analyzed and compared. The stock solutions of free MG and MG-MSN were prepared by adding five mg of MG in 5 ml of distilled water and stored in dark conditions. A diode laser with a wavelength of 670 nm was used for irradiation. The energy fluence was calculated using the formula described below (Misba, Kulshrestha & Khan, 2016)

\begin{eqnarray*}& & \text{Energy fluence}=\mathrm{PD}\times \mathrm{T}. \end{eqnarray*}

Where, PD; Power density and T: time

\begin{eqnarray*}& & \text{Power density (PD)}=\text{Output power of light source (mW)}/\text{irradiated area}({\mathrm{cm}}^{2}). \end{eqnarray*}

The diameter of the well (seven mm) was the same as the diameter of the irradiated area and energy fluence was calculated as 97.65 J cm^−2^ when exposed for 5 min. The output power used for experiments was 125 mW. A final concentration of 50 µg/ml (MSN, MG, and MG-MSN) was used in all the experiments. The illumination time was found to be 5 min for both the test bacteria.

### Antimicrobial photodynamic therapy

The aPDT of planktonic cells was performed as per the procedure of Hsieh et al. (2014) with slight modifications. Bacterial cultures were adjusted to a McFarland standard of 0.5 by measuring the absorbance at 600 nm. Eppendorf tubes containing 250 µl of culture was treated with 50 µg/mL of MSN, MG and MG-MSN. One tube containing culture was maintained as control and all tubes incubated in dark for 3 h. After incubation, centrifugation was performed for 5 min at 10,000 rpm to separate the unbound dye and MSN. The pellet was collected and dissolved in sterile PBS. About 200 µl of solution was pipetted out in 96 well microtiter plates. Two experimental setups each containing a control, free compounds (MG, MSN) and MG-MSN were prepared for both dark and light treatments. One experimental set was irradiated using a laser of wavelength, 670 nm for 5 min. Another setup was incubated in dark. After irradiation, all samples from two experimental setup were 10 fold diluted The dilutions of 10^−3^ and 10^−4^ were plated and incubated at 37 °C for 48 h. The colonies grown on plates were counted as CFU/ml and represented as percentage reduction in cells after comparing with the control CFUs.

### Detection of ROS

Endogenous ROS generation during aPDT was detected using 2′, 7′ dichlorofluorescein-diacetate (DCFH-DA) (Wang et al., 2016). Each sample was treated with five µM of DCFH-DA after adjusting both the test cultures to 0.5 McFarland’s standard and incubated in dark for 10 min. The cells were treated with MSN, MG, MG-MSN and these samples and untreated control samples were pre-incubated in the dark for 3 h. One experimental set-up was exposed to 670 nm laser light. Another set-up was used for dark incubation. The intensity of fluorescence was studied by excitation at 485 nm using a fluorescence spectrofluorometer (Make: Jobin Yvon, Model: Fluorolog-FL3-11).

### Inhibition of biofilm formation

Biofilm inhibition was determined as per the protocol of Sánchez et al. (2016). Diluted bacterial cultures were pipetted into 96 well microtiter plates and treated with MSN, MG, and MG-MSN. The plates were pre-incubated in the dark for 3 h with the respective control blanks. Then all the samples were irradiated with laser light for 5 min and incubated overnight at 37 °C for 18 h. Similar experiments were performed in dark conditions as well. This allowed the formation of biofilms. The unadhered cells were washed out using sterile PBS. Two hundred microliters of crystal violet solution (0.1%) was dispensed to each well and left undisturbed for 15 min. Then, the wells were washed thrice using sterile PBS. About 100 µl of 95% ethanol was added in order to extract the cell bound crystal violet and the OD was measured at 595 nm. The inhibition of biofilm formation was represented as percentage reduction by comparing with the control using the following formula

\begin{eqnarray*}\text{%} \text{Biofilm inhibition}=(\text{Absorbance in control at 595 nm {\ndash} absorbance in test at 595 nm})/\text{Absorbance in control at 595 nm}\times 100. \end{eqnarray*}

### Cell viability assay

Cellular metabolism was determined using the tetrazolium chloride (TTC) assay (Misba, Zaidi & Khan, 2017). Overnight test bacterial cultures were pre-incubated in the dark for 3 h after the addition of MSN, MG, and MG-MSN (50 µg/ml). The samples were irradiated for 5 min and incubated at 37 °C for 18 h. A similar set-up was maintained in dark condition as well. One hundred microliters of TTC (0.5%) was added to all samples after washing with sterile PBS and then incubated for 30 min. The live cells will convert TTC to formazon. The absorbance was measured at 490 nm. The reduction in the cell viability after irradiation was determined using the formula below.

\begin{eqnarray*}\text{%} \text{Reduction in the cell viability}=(\text{Absorbance in control at 490 nm}-\text{Absorbance in}\nonumber\\\displaystyle \text{test at 490 nm})/\text{Absorbance in control at 490 nm}\times 100. \end{eqnarray*}

### Quantification of exopolysaccharides (EPS)

Congo red (CR) binding assay was used for investigating reduction in the EPS production (Misba, Zaidi & Khan, 2017). Sterile brain heart infusion broth supplemented with 1% glucose was added to the test cultures after adjusting to the McFarland’s standard (1.5  × 10^8^ CFU/ml). The samples were treated with MSN, MG, and MG-MSN (50 µg/ml each) and pre-incubated in the dark. One experimental setup was then exposed to laser for 5 min and other experimental set was left undisturbed in dark. All the samples were incubated at 37 °C for 18 h. Then, the medium was washed off with sterile PBS twice. Fresh medium (100 µL) containing 0.5 mM of CR (50 µL) was added in treated wells as well as blank and control. The microtiter plates were kept as such for 2 h and then the cells were separated by centrifugation for 5 min at 10,000 rpm. The supernatant was withdrawn and abosrbance was recorded at 490 nm. Optical density recorded for blank CR was used to determine the difference in the absorbance values of treatments. The reduction in EPS was quantified using the formula given below:

\begin{eqnarray*}\text{%} \text{Reduction in EPS}=(\text{Absorbance in control at 490 nm}-\text{Absorbance in test at 490 nm})/\nonumber\\\displaystyle \text{Absorbance in control at 490 nm}\times 100. \end{eqnarray*}

### Microscopic observation of biofilms

Confocal Laser Scanning Microscpy (CLSM) was performed to confirm the antibiofilm activity of MG-MSN against the test strains after aPDT (Viszwapriya et al., 2016). The biofilm was established by culturing test bacteria in 12-well polystyrene microtiter plates with glass coverslip. The plate was covered and sealed with Parafilm and incubated for 48 h at 37 °C. After treating the established biofilms with MSN, MG, and MG-MSN, the light and dark treatments were performed similarly as described in the previous assays. After the treatment, all the samples were incubated at 37 °C for 48 h. Unadhered cells from light-treated and dark-incubated samples were removed with sterile PBS after 48 h. The adhered biofilm that remained after the washing step was dual stained (ethidium bromide and acridine orange) and left for 15 min at room temperature. Biofilms were visualized after aPDT through the emission of fluorescence through a CLSM.

### Statistical analysis

Three independent experiments of each treatment were performed *in vitro*. The results obtained were compared with their respective controls and represented as mean  ± standard deviation of triplicate results. One-way analysis of variance (ANOVA) was used to analyze the obtained data. The obtained data having *p*-values ≤ 0.05 were treated as statistically significant and recorded.

## Results

### Characterization of MG-MSN

**Figure 1:**
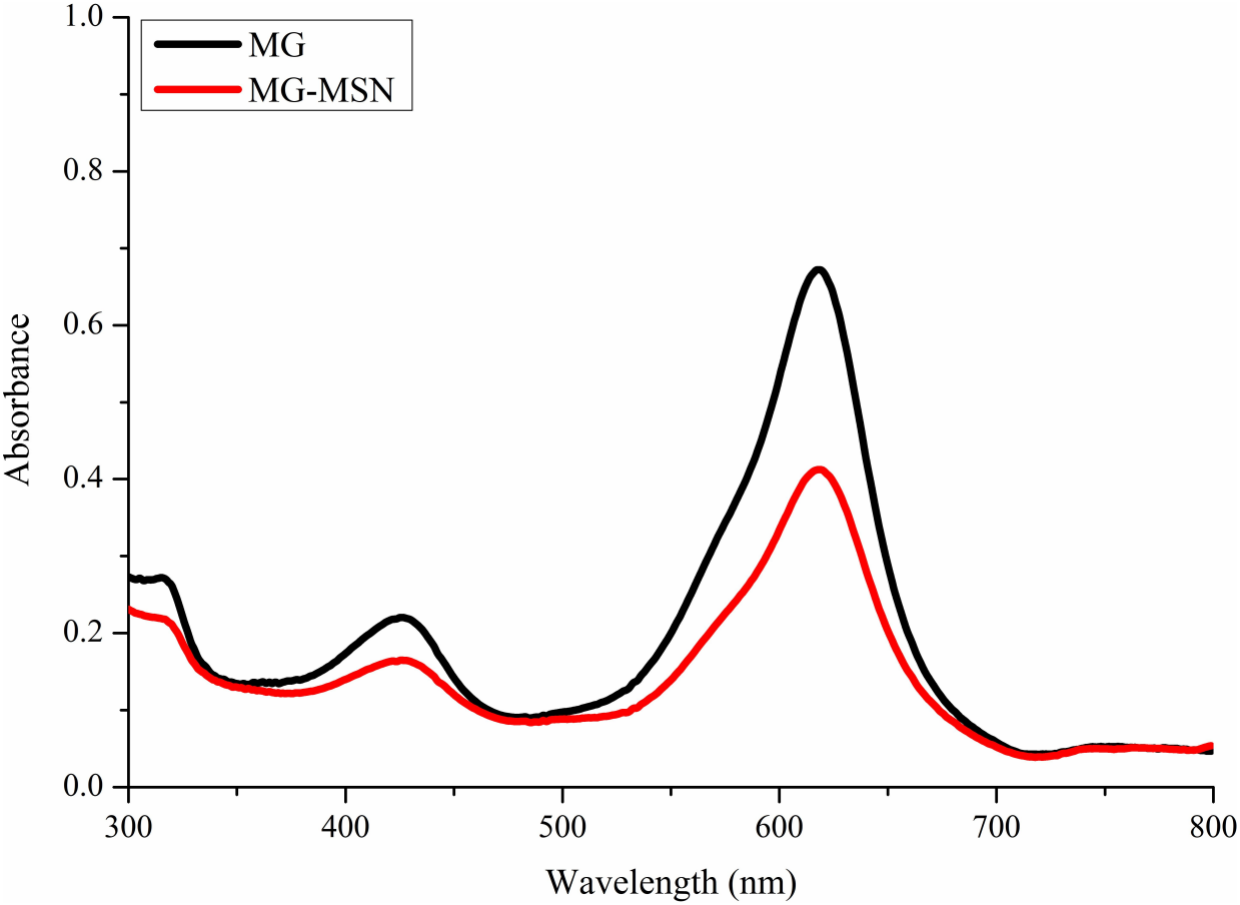
UV-vis absorption spectra of free MG and MG-MSN.

The UV-Visible absorption spectra of MG and MG-MSN were recorded (Fig. 1). The characteristic surface plasmon resonance absorption peak for MG was formed at 618 nm. An identical and overlapping surface plasmon peak was observed for MG-MSN suggesting that the encapsulation of MG had occurred.

**Figure 2:**
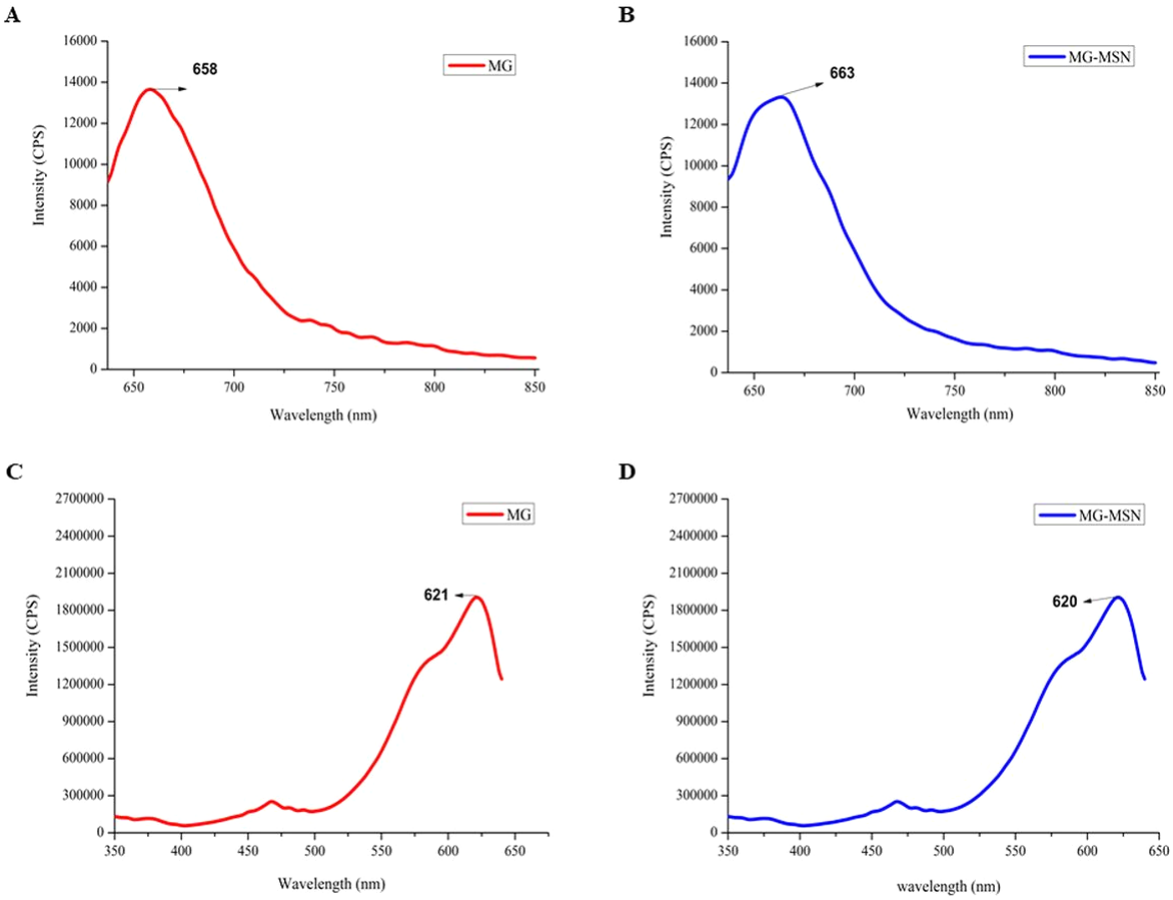
Analysis of fluorescent spectrum of MG and MG-MSN. (A) and (B) represents the photoluminescence emission spectra and (C) and (D) represents photoluminescence excitation spectra.

Fluorescence spectra of MG and MG-MSN are shown in Fig. 2. Photoluminescence emission spectrum of MG is represented in Fig. 2A with maximum emission at 660 nm. Similarly, in Fig. 2B, the emission spectrum of MG-MSN showed maximum emission at the corresponding wavelength of MG. Furthermore, the excitation spectra of MG and MG-MSN depicted in Figs. 2C and 2D, respectively, showed maximum intensity at 620 nm. The trends observed in both emission and excitation spectra revealed that MG has been encapsulated by MSN.

**Figure 3:**
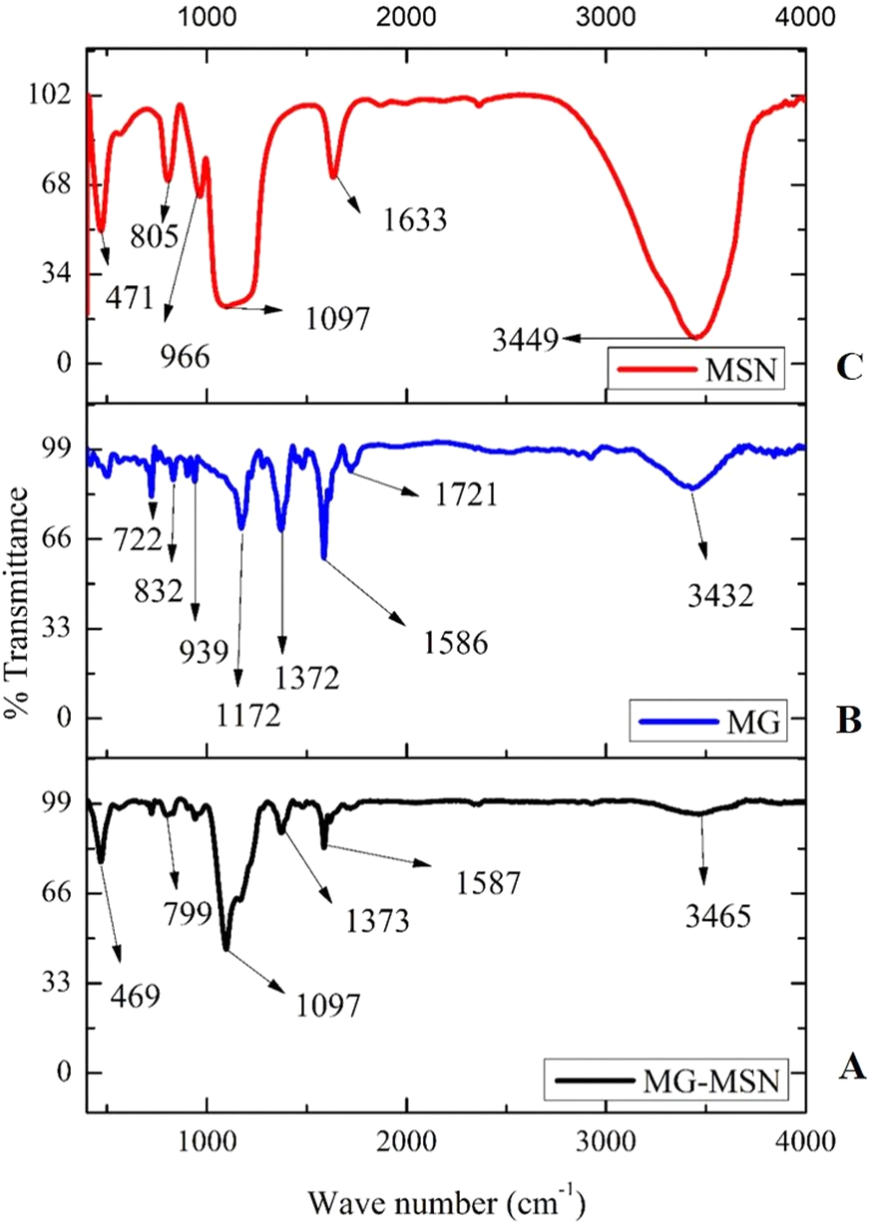
FTIR analyses of MG encapsulated MSN, in comparison with MSN and free MG.

Functional groups present in MSN, MG, and MG-MSN are clearly depicted in Fig. 3. Stretching vibrations of Si-O-Si, Si-OH, and Si-O are represented by the sharp peaks at 800, 959, and 1,090 cm^−1^ in MSN. The OH vibration is observed at 3,449 cm^−1^. For MG, specific peaks between 500 and 1,500 cm^−1^represent monodistributed and paradistributed benzene rings. The strong peak at 1,586 cm^−1^corresponds to the C=C stretching vibration and the one at 1,172 cm^−1^ represents C-N stretching vibration. The peak at 3,432 cm^−1^ is due to O-H or N-H vibration. The FTIR spectrum of MG-MSN also clearly specifies the successful encapsulation.

**Figure 4:**
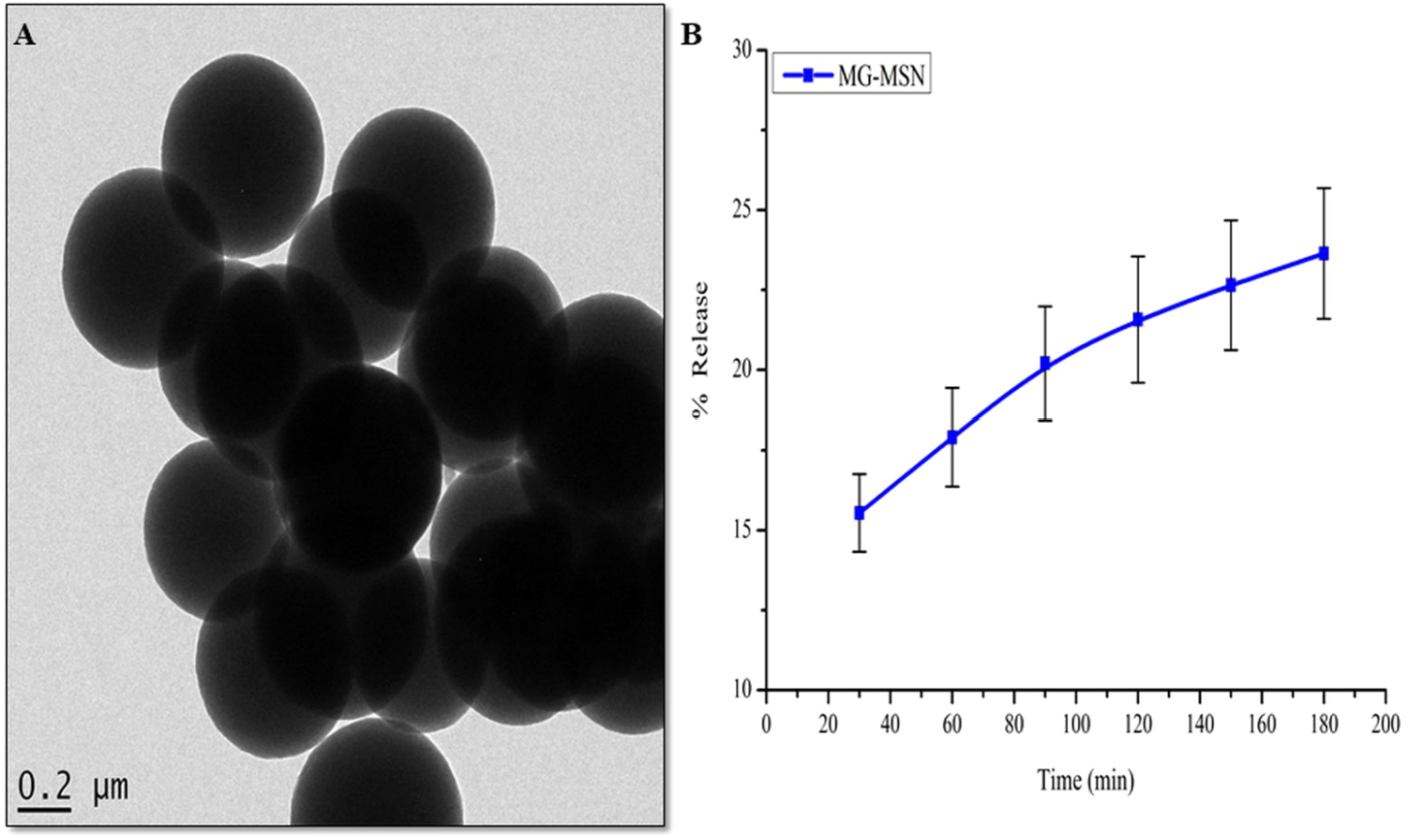
(A) HR-TEM micrograph of MG-MSN and (B) *in vitro* release profile of MG from MG-MSN. Error bars represent the standard deviation.

Figure 4A represents the HRTEM micrograph of MG-MSN. It depicts the shape and size of MG-MSN. The image represents spherical shaped and evenly distributed MG-MSN with an average size of 500 nm.

### Loading capacity (LC) of dye and entrapment efficiency (EE)

The entrapment efficiency of MG was determined as 60.07 ± 3.2%. The loading capacity of MSN was calculated as 12.01 ± 1.1%. It was found that the loading capacity of MSN is influenced by its porous structure and shape.

### Release profile of MG

Release profile of MG-MSN is depicted in Fig. 4B. The release percentages of MG at the 30th and 180th min were 15.54 ± 1.21 and 23.63 ± 2.04 respectively. The results suggested a gradual slow release of the dye from MG-MSN and this is beneficial for effective antimicrobial therapy.

### Bacterial uptake study

Bacterial uptake study suggests that MG-MSN enhanced the accumulation of a high concentration of MG inside the bacterial cells. The uptake after the 180th min in *E. coli* was found to be 51.40 ± 3.644% for MG-MSN and 30.49 ± 1.62% for MG. Similarly, in *S. aureus,* the uptakes at 180th min for MG-MSN and MG were found to be 68.87 ± 2.02% and 35.38 ± 1.44%, respectively. The percentage uptake profiles of both the test strains at different time intervals are represented in Table 1. The percentage uptake of MG-MSN was found be higher in the Gram positive bacterium, *S. aureus* than Gram negative *E. coli.*

**Table 1:** Cellular uptake profile of MG and MG MSN by *E. coli* and *S. aureus* at different time intervals.

Organism	*E. coli*		*S. aureus*	
Time (min)	**MG (%)**	**MG-MSN (%)**	**MG (%)**	**MG-MSN (%)**
30	6.07 ± 0.008	13.67 ± 0.30	6.75 ± 0.16	6.04 ± 2.90
60	6.12 ± 0.007	16.40 ± 0.46	16.35 ± 0.42	11.61 ± 0.82
90	8.22 ± 0.79	20.41 ± 0.76	18.20 ± 0.23	13.81 ± 1.16
120	13.98 ± 0.14	29.36 ± 1.08	20.87 ± 2.09	35.30 ± 1.05
150	18.85 ± 0.316	37.09 ± 5.51	33.03 ± 1.18	45.98 ± 1.34
180	30.49 ± 1.62	51.40 ± 3.60	35.38 ± 1.44	68.87 ± 2.02

### Antimicrobial photodynamic therapy

**Figure 5:**
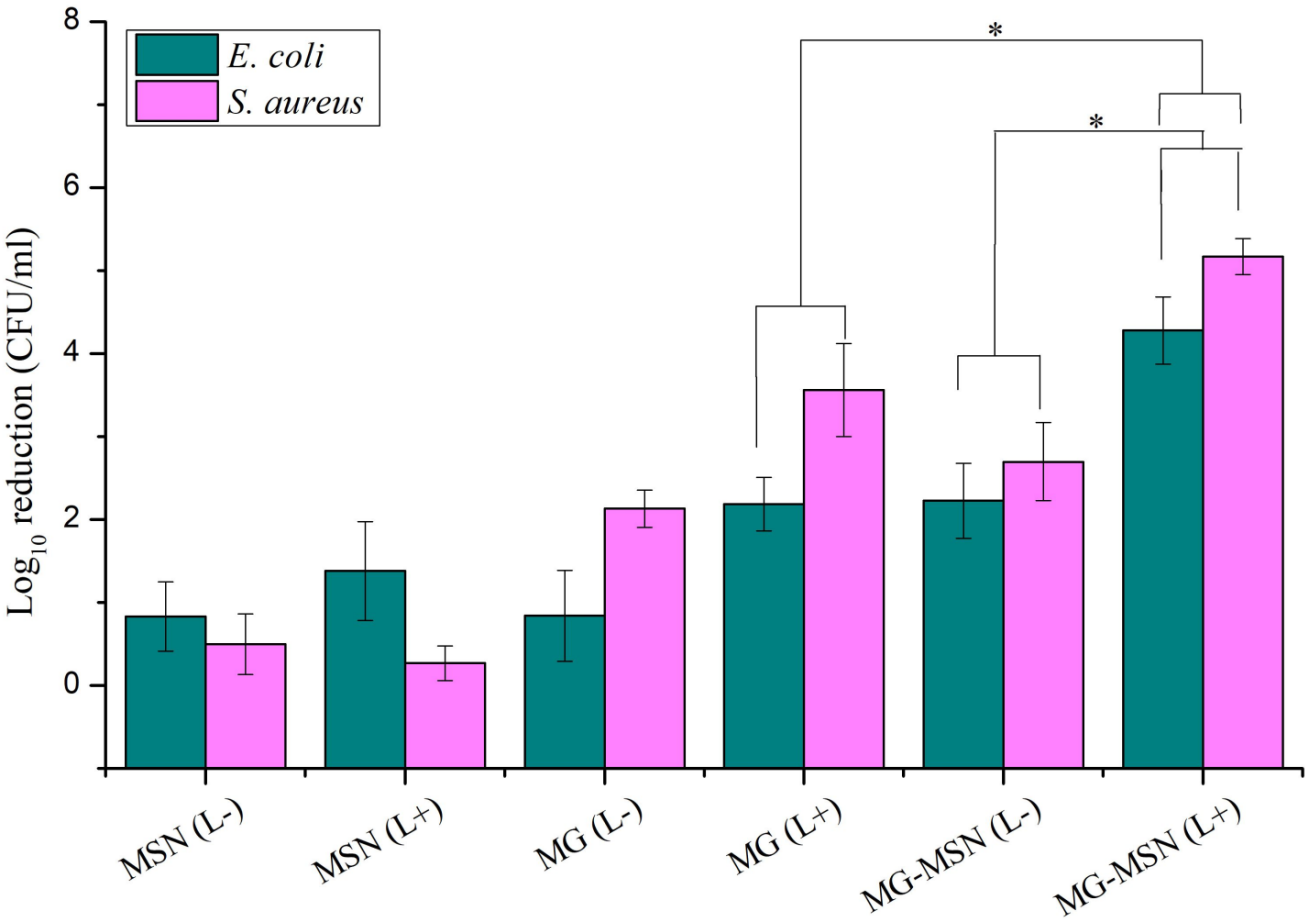
Effect of antimicrobial photodynamic inactivation on planktonic cells of *E. coli* and *S. aureus* using MSN, MG and MG-MSN. Error bars represent the standard deviation and * Represents the significance level at *p* < 0.05.

Antimicrobial photodynamic therapy of test bacteria using MG-MSN is represented as log_10_ reductions (Fig. 5). The antibacterial efficacy of MG-MSN was found to be greater against *S. aureus* than *E. coli*. The log_10_ reduction in *E. coli* after light treatment with MG-MSN was found to be 4.27 ± 0.40 and with MG it was 2.18 ± 0.32. In the case of *S. aureus*, after aPDT using MG-MSN, the reduction in the number of cells was found to be 5.16 ± 0.21 and with MG the reduction obtained was 3.56 ± 0.56. Treatment with MG-MSN was more effective against both the test strains compared to the free dye.

### Detection of ROS

**Figure 6:**
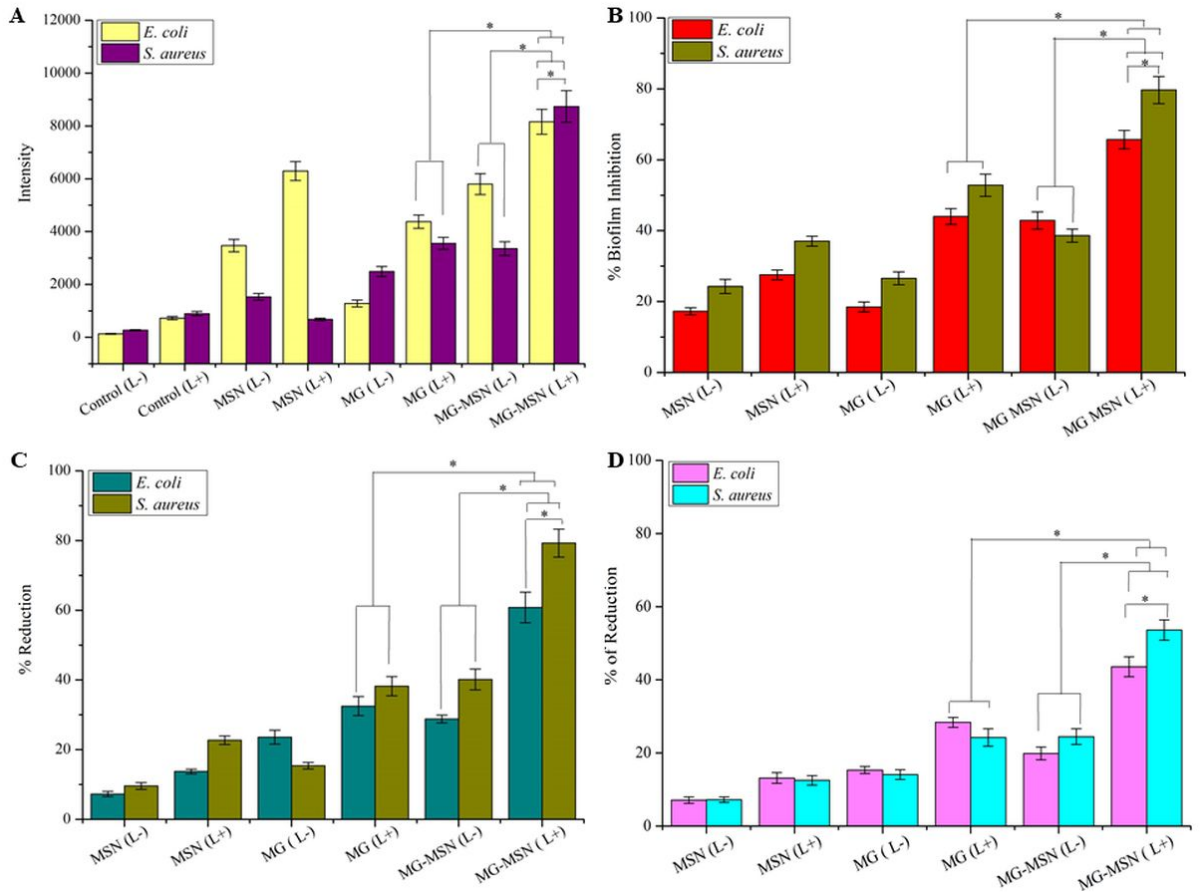
The bar graphs were representing effect of aPDT on test bacteria in their biological activity; (A) ROS generation, (B) biofilm inhibition, (C) reduction in cellular viability (D) percentage reduction on EPS formation of *E. coli* and *S. aureus* by aPDT treatment using MSN, MG and MG-MSN, respectively. Error bars represent the standard deviation and * Represents the significance level at *p* < 0.05.

The most crucial and fundamental step in aPDT is the production of ROS from light activated PS. Photoinduced MG-MSN produced ROS, detected as increased fluorescence intensity. This ROS would attack different biological constituents including nucleic acids, protein and lipids. Non-specific interaction of ROS with biomolecules provided significant inhibition of both planktonic cells and biofilms. ROS generation was measured using the spectrofluorometer and the intensities obtained are graphically represented in Fig. 6A. ROS generation in MG-MSN-treated samples was ten times more when compared to control. When compared to free MG, the ROS generated was significantly higher in samples treated with MG-MSN.

### Biofilm inhibition assay

Novel antibiofilm strategies especially aPDT had been recently gained interest in combating infections caused by bacterial biofilms resistant to antibiotics. The antibiofilm efficacy of MG-MSN was studied using crystal violet dye. The biofilm inhibition in *E. coli* after treatment with MG-MSN was 65.68 ± 2.62% and with free MG it was 44.01 ± 2.21% (Fig. 6B). In *S. aureus*, the biofilm inhibition was found to be 79.66 ± 3.82% and 52.82 ± 3.12% in MG-MSN and free MG treated samples, respectively. MG-MSN treated samples were more susceptible and the reduction in biofilm formation was higher in them than the MG treated samples.

### Cell viability assay

Metabolic activity of a cell is the true representation of its viability and was determined using TTC. Viability of test bacteria after aPDT was significantly reduced upon treatment with MG-MSN. In *E. coli*, the percentage reduction in MG-MSN and MG treated samples was found to be 60.78 ± 4.39% and 32.50 ± 2.72% respectively (Fig. 6C). *S. aureus* cultures showed more susceptibility than *E. coli* and the reduction in live cells was found to be 79.27 ± 4.01% and 38.23 ± 2.76% respectively after MG-MSN and MG treatment.

### Reduction of EPS

EPS are the major components of biofilm and contribute at least 90% by weight of the total biofilm content and facilitate structural complexity and strength of the biofilm, thus EPS is considered as a first line of defense against diffusion of antibiotics and restricts the penetration of PS. The reduction in EPS production could reduce the strength of the biofilm simplifying the treatment process. *E. coli* exhibited a reduction of 43.57 ± 2.75% in MG-MSN treated samples while MG treated samples gave a reduction of 28.36 ± 1.37% (Fig. 6D). Production of EPS was reduced to 53.60 ± 2.77% in MG-MSN-treated *S. aureus*. The reduction was 24.22 ± 2.40% in MG-treated *S. aureus*.

### CLSM analysis

**Figure 7:**
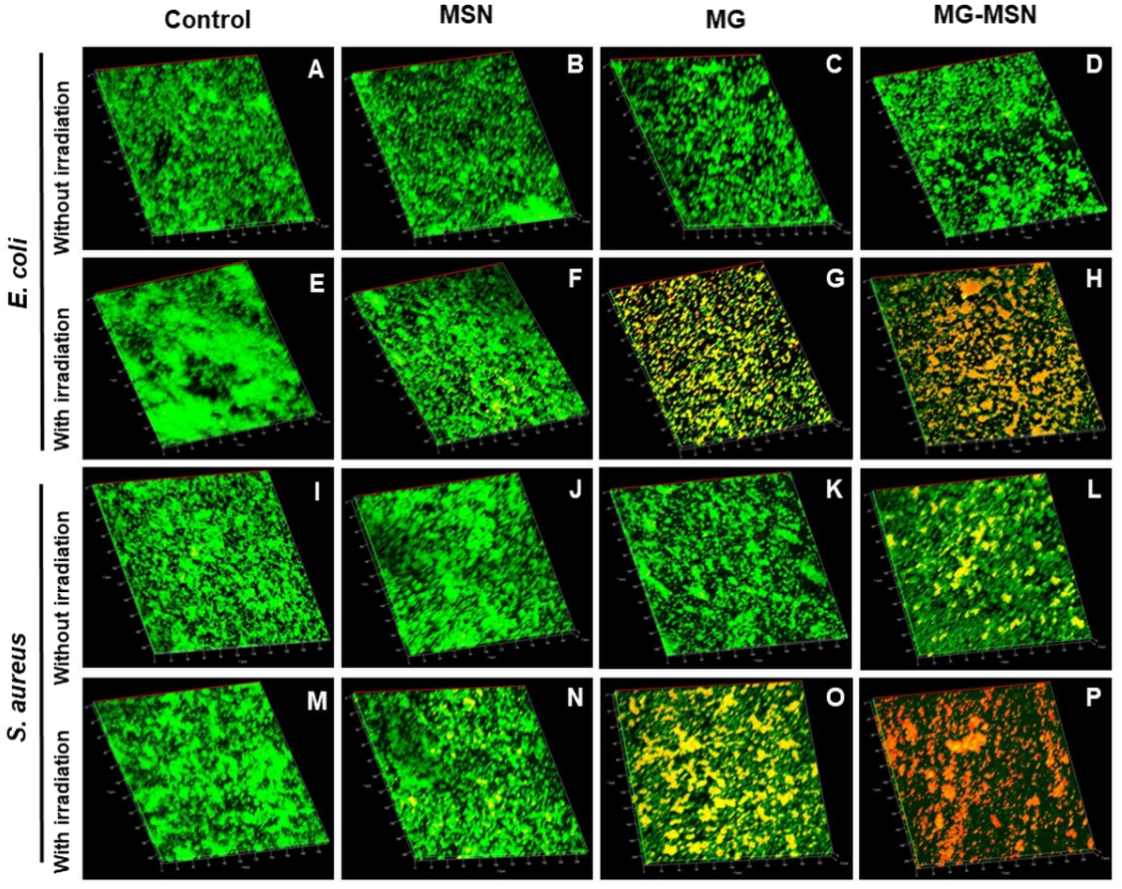
CLSM images demonstrating the aPDT efficacy on biofilms of test bacteria after stained with acridine orange and ethidium bromide. The micrographs represent the live (green) and dead (red) cells of both *E. coli* and *S. aureus* of the untreated control without irradiation (A & I), MSN treated without irradiation (B & J), MG treated without irradiation (C & K), MG-MSN treated without irradiation (D & L), the untreated control with irradiation (E & M), MSN treated with irradiation (F & N), MG treated with irradiation (G & O), MG-MSN treated with irradiation (H & P), respectively.

Antibiofilm activity of MG-MSN on the test cultures was analyzed by CLSM and the micrographs obtained are presented in Fig. 7. Green fluorescent mat obtained in the control represents the live cells whereas the red fluorescence in treated samples indicates dead cells. More red fluorescence was detected in bacterial samples treated with MG-MSN exposed to light. This suggests that the nano encapsulated MG-MSN is more active than the free MG.

## Discussion

Antimicrobial photodynamic therapy is a novel strategy that utilizes light energy of a particular wavelength and a photosensitizing molecule to eliminate bacteria (Thakuri et al., 2011). A number of PSs are reported as antimicrobial photosensitizing agents against a wide range of microorganisms that include bacteria, yeast, viruses, and pathogenic algae. Cationic PSs are more effective in the treatment of both Gram positive and Gram negative bacteria and are widely used for their broad spectrum of action (Carvalho et al., 2009). The ROS generated during the aPDT process by the light activated PS will react with almost all the biomolecules and cellular components and this causes the death of bacteria. *S. aureus* and *E. coli* are major causes of nosocomial infections (Hanakova et al., 2014). The development biofilms is a major advantage to these microorganisms that helps them to evade almost all antibiotics (Ragàs, Agut & Nonell, 2010). Recently the antimicrobial photodynamic potential of riboflavin was reported against clinically isolated, *E. coli* suggesting its potential in the treatment of nosocomial infections (Khan et al., 2019).

In the present study, we focused on MG encapsulated on MSN to augment the photodynamic effect, and antimicrobial and antibiofilm efficacies of MG-MSN against Gram positive and Gram negative bacteria were evaluated. MSN loaded with MG was employed as a carrier to deliver the MG to the test pathogens which enhanced the antimicrobial performance. The product obtained after encapsulation was light green colored and was stored in the dark. Encapsulation of dye on nanoplatforms was confirmed by employing various characterization techniques. UV-Vis spectra of MG and MG-MSN were analyzed and both the spectra were overlapping having a maximum absorbance at 618 nm. The results of UV-Vis spectra were in good agreement with previous reports (Huang et al., 2015). Fluorescence spectroscopic analysis also confirmed the encapsulation as the emission and excitation peaks of MG and MSN were obtained in the same range.

Functional groups present in MSN, MG and MG-MSN were recorded using FTIR. FTIR spectrum of MSN was observed with strong peaks at 800, 959, and 1090 cm^−1^. The stretching vibrations of Si-O-Si is indicated by the peak at 800 cm^−1^ and Si-OH group at 959 cm^−1^. Another peak of MSN corresponding to its Si-O stretching vibration was observed at 1,090 cm^−1^. The OH vibration of MSN was indicated the peak at 3,449 cm^−1^. This was in agreement with the previous studies (Khosraviyan et al., 2016). FTIR analysis of MG was also in agreement with previous studies (Cheriaa et al., 2012; Bouaziz et al., 2017; Deniz & Kepekci, 2017). In case of MG-MSN, the peaks observed for MG and MSN were present, confirming the presence of both the dye and the nanoparticles. The HRTEM micrograph depicted that shape, size, dispersity and porosity of MSN held a pivotal role in good dye loading and encapsulation (Mehmood et al., 2017). The entrapment efficiency of MG on MSN was found to be 60.07 ± 3.2% and the loading capacity of MSN was calculated as 12.01 ± 1.1%. It was found that the loading capacity of MSN is influenced by its porous structure and shape as supported by the previous studies (Wang et al., 2011; Zou et al., 2013).

The bacterial uptake of MG-MSN at the 180th min was about 51.40% in *E. coli* and 68.87% in *S. aureus* while the uptake of free MG was lower in *E. coli* (30.9%) and *S. aureus* (35.38%)*.* This clearly revealed that the nano platform enhanced the percentage uptake significantly and was comparable with published data (Yan et al., 2018). The release profile was found to be slow and gradual with time. The advantage of sustained release is that aPDT can be repeated for a number of times to completely eradicate the pathogen without the formation of resistant strains (Usacheva et al., 2016). The release profile showed a sustained release of dye from the nanoparticle was having an enhanced activity with a shorter incubation time. Here, sustained release of MG might facilitate enhanced antibacterial activity with repeated photodynamic therapy after single administration of compounds.

aPDT on planktonic cells of both the test strains suggested a significant reduction in the MG-MSN treated bacteria after light exposure for 5 min. The control groups without light irradiation had less effect on cells and revealed that the PS has no dark toxicity. Gram positive bacteria showed more susceptibility to aPDT than the Gram negative bacteria, in agreement with a previous study (Tawfik, Alsharnoubi & Morsy, 2015). This variation in susceptibility of Gram negative and Gram positive bacteria can be explained by physiological differences. Gram positive bacteria have a cytoplasmic membrane covered by a simple cell wall that allows high internalization of PSs into their bacterial cells. The outer envelope of Gram negative bacteria is relatively complex, poorly facilitating internalization of the PSs. This variation in accumulation of PSs inside the two types of bacterial cells, gives relative differences in their susceptibility indexes (Misba, Zaidi & Khan, 2017). Free dye has less of an effect on planktonic cells, and the results revealed that the use of nanovehicle MSN improved the activity of MG-MSN because of good binding, uptake, and controlled release. The most crucial and fundamental step in aPDT is the production of ROS from light activated PS. ROS generation was measured using the spectrofluorometer and the intensities obtained are graphically represented in Fig. 6A. ROS generation in MG-MSN-treated samples was ten times more when compared to the control. When compared to free MG, the ROS generated was significantly higher in the samples treated with MG-MSN. This was due to the internalization of a high concentration of MG inside the bacterial cells with MG-MSN as compared to free MG (Table 1). *S. aureus* was found to be more susceptible than *E. coli* because of the substantially increased production of ROS as a consequence of accumulation of a high concentration of MG inside the Gram positive bacteria when compared with Gram negative bacteria due to the difference in their cytoplasmic physiology (Dwivedi et al., 2014).

The antibiofilm efficacy of MG-MSN was studied using crystal violet staining assay. Biofilm architecture is protected by a self-produced polymeric matrix consisting of proteins, polysaccharides, extracellular DNA, and rhamnolipids (Hu et al., 2018). The ROS produced from MG interacted with these biomolecules present in the biofilms and ultimately disturbed the matrix. Biofilm inhibition assay results depicted in Fig. 6B reveal that the reduction in biofilm formation is more in *S. aureus* compared to *E. coli* (Singh et al., 2016). The viability of cells after aPDT in both the test cultures diminished significantly. Reduced EPS production also suggested the enhanced activity of MG-MSN on both the test cultures. Biofilm inhibition efficacy was further confirmed by microscopic analysis using CLSM, which revealed that MG-MSN treated samples had more dead cells as indicated by red fluorescence. The results concur with those form previous studies (Misba, Zaidi & Khan, 2017).

## Conclusion

The present study confirmed the enhanced antimicrobial and anti-biofilm activities of MG-MSN against the two tested bacterial cultures using aPDT. The antibacterial activity was significant against both the test bacteria, but the Gram positive bacterium *S. aureus* was found to be more susceptible than Gram negative *E. coli*. Antibiofilm efficacy of the nanocomposite material was also studied and confirmed through different assays (inhibition of biofilm by crystal violet, quantification of EPS, and TTC assay for cell viability). CLSM micrographs confirmed the improved activity. To the best of our knowledge, this is the first study on aPDT using MG-MSN against different groups of biofilm producing, drug resistant strains of bacteria such as *E. coli* and *S. aureus*. This study can be also extended to eradicate these strains from superficial localized infections and from medical appliances and thus help to prevent nosocomial infections.

##  Supplemental Information

10.7717/peerj.7454/supp-1 Data S1 Raw data Raw data for Antimicrobial photodynamic therapy, Detection of ROS, Biofilm inhibition, Cell viability assay and Reduction of exopolysaccharides.

## Additional Information and Declarations

### Data Availability

The following information was supplied regarding data availability: The raw data are available as Supplemental File.
