# Supplementary material for: Antimicrobial photodynamic therapy on Staphylococcus aureus and Escherichia coli using malachite green encapsulated mesoporous silica nanoparticles: an in vitro study
Source: PeerJ. 2019 Sep 12;7:e7454. doi: 10.7717/peerj.7454 (PMC6745189; doi:10.7717/peerj.7454)
Supplement: Data S1 — Raw data for Antimicrobial photodynamic therapy, Detection of ROS, Biofilm inhibition, Cell viability assay and Reduction of exopolysaccharides. [file peerj-07-7454-s001.docx]

**Antimicrobial photodynamic therapy on Staphylococcus aureus and Escherichia coli using malachite green encapsulated mesoporous silica nanoparticles – an in vitro study**

**Antimicrobial photodynamic therapy**

| **Experimental conditions** | ***E. coli*** | | | ***S. aureus*** | | |
| --- | --- | --- | --- | --- | --- | --- |
|  | 1 | 2 | 3 | 1 | 2 | 3 |
| **Control (L^-^)** | 3.8x10^7^ | 6.8x10^7^ | 8.1x10^7^ | 6.8x10^7^ | 7.5x10^7^ | 9.3x10^7^ |
| **Control (L^+^)** | 1.1x10^8^ | 4.4x10^7^ | 5.6x10^7^ | 8.7x10^7^ | 9.9x10^7^ | 6.2x10^7^ |
| **MSN (L^-^)** | 8.9x10^6^ | 1.9x10^7^ | 3.9x10^6^ | 3.2x10^7^ | 8.9x10^6^ | 5.0x10^7^ |
| **MSN (L^+^)** | 0.1x10^7^ | 5.8x10^6^ | 3.3x10^6^ | 5.7x10^7^ | 5.6x10^7^ | 1.9x10^7^ |
| **MG (L^-^)** | 4.6x10^6^ | 0.3x10^7^ | 4.4x10^7^ | 7.2x10^5^ | 0.3x10^6^ | 8.4x10^5^ |
| **MG (L^+^)** | 8.4x10^5^ | 5.4x10^5^ | 2.3x10^5^ | 0.6x10^4^ | 2.5x10^4^ | 5.5x10^4^ |
| **MG-MSN (L^-^)** | 7.2x10^5^ | 1.7x10^5^ | 3.5x10^5^ | 0.4x10^5^ | 2.3x10^5^ | 4.1x10^5^ |
| **MG-MSN (L^+^)** | 2x10^3^ | 4.5x10^3^ | 6.3x10^3^ | 5.6x10^2^ | 4.2x10^2^ | 7.1x10^2^ |

*Values are expressing in CFU/ml

*(L^-^) – Without irradiation; (L^+^) – With irradiation

**Detection of ROS**

| **Experiment Condition** | ***E. coli*** | | | | ***S. aureus*** | | | |
| --- | --- | --- | --- | --- | --- | --- | --- | --- |
|  | 1 | 2 | 3 | Mean | 1 | 2 | 3 | Mean |
| **Control (L^-^)** | 116.51 | 123.76 | 146.41 | **128.89 ±** 15.59 | 244.18 | 287.16 | 266.72 | **266.02 ± 21.49** |
| **Control (L^+^)** | 683.53 | 793.23 | 691.03 | **722.59 ± 61.28** | 985.04 | 859.23 | 835.62 | **893.29 ± 80.32** |
| **MSN (L^-^)** | 3589.50 | 3623.28 | 3198.86 | **3470.54 ± 235.89** | 1654.97 | 1538.75 | 1399.87 | **1531.19 ± 127.72** |
| **MSN (L^+^)** | 6061.78 | 6121.10 | 6708.76 | **6297.21 ± 357.63** | 636.04 | 723.65 | 669.97 | **676.55 ± 44.17** |
| **MG (L^-^)** | 1182.10 | 1424.76 | 1231.98 | **1279.61 ± 128.14** | 2603.65 | 2265.82 | 2591.15 | **2486.87 ± 191.54** |
| **MG (L^+^)** | 4627.20 | 4376.54 | 4127.37 | **4377.04 ± 249.91** | 3544.16 | 3781.98 | 3325.67 | **3550.60 ± 228.22** |
| **MG-MSN (L^-^)** | 5988.59 | 6071.21 | 5344.56 | **5801.45 ±**  397.83 | 3302.14 | 3126.98 | 3632.24 | **3353.79 ± 256.55** |
| **MG-MSN (L^+^)** | 8596.744 | 7658.92 | 8212.198 | **8155.95 ±**  471.43 | 9324.91 | 8764.36 | 8127.92 | **8739.06 ± 598.89** |

*Values of fluorescence intensity (485/535 nm)

*(L^-^) – Without irradiation; (L^+^) – With irradiation

**Biofilm inhibition**

| **Experiment Condition** | ***E. coli*** | | | | ***S. aureus*** | | | |
| --- | --- | --- | --- | --- | --- | --- | --- | --- |
|  | 1 | 2 | 3 | Mean | 1 | 2 | 3 | Mean |
| **MSN (L^-^)** | 16.20 | 17.27 | 18.19 | **17.22 ± 0.99** | 24.56 | 22.16 | 26.11 | **24.28 ± 1.99** |
| **MSN (L^+^)** | 26.39 | 27.12 | 29.01 | **27.51 ± 1.35** | 37.31 | 35.49 | 38.24 | **37.01 ± 1.40** |
| **MG (L^-^)** | 18.26 | 19.96 | 17.16 | **18.46 ± 1.40** | 25.23 | 25.82 | 28.63 | **26.56 ± 1.81** |
| **MG (L^+^)** | 41.79 | 44.03 | 46.22 | **44.01 ± 2.21** | 56.20 | 50.05 | 52.21 | **52.82 ± 3.12** |
| **MG-MSN (L^-^)** | 40.42 | 45.31 | 42.88 | **42.87 ± 2.44** | 36.47 | 39.30 | 39.98 | **38.58 ± 1.85** |
| **MG-MSN (L^+^)** | 63.65 | 68.64 | 64.75 | **65.68 ± 2.62** | 82.83 | 80.75 | 75.42 | **79.66 ± 3.82** |

*Values are expressing the percentage of inhibition

*(L^-^) – Without irradiation; (L^+^) – With irradiation

**Cell viability assay**

| **Experiment Conditions** | ***E. coli*** | | | | ***S. aureus*** | | | |
| --- | --- | --- | --- | --- | --- | --- | --- | --- |
|  | 1 | 2 | 3 | Mean | 1 | 2 | 3 | Mean |
| **MSN (L^-^)** | 6.44 | 7.75 | 7.66 | **7.29 ± 0.73** | 9.02 | 10.71 | 9.03 | **9.59 ± 0.97** |
| **MSN (L^+^)** | 13.06 | 13.77 | 14.40 | **13.74 ± 0.67** | 21.35 | 22.93 | 23.83 | **22.71 ± 1.25** |
| **MG (L^-^)** | 21.60 | 23.52 | 25.59 | **23.57 ± 1.99** | 14.51 | 15.26 | 16.40 | **15.39 ± 0.95** |
| **MG (L^+^)** | 30.25 | 31.72 | 35.52 | **32.50 ± 2.72** | 39.10 | 35.13 | 40.46 | **38.23 ± 2.76** |
| **MG-MSN (L^-^)** | 28.41 | 27.93 | 30.03 | **28.79 ± 1.10** | 37.77 | 43.52 | 39.17 | **40.15 ± 2.99** |
| **MG-MSN (L^+^)** | 57.06 | 65.63 | 59.66 | **60.78 ± 4.39** | 82.90 | 74.96 | 79.97 | **79.27 ± 4.01** |

*Values are expressing the percentage of inhibition

*(L^-^) – Without irradiation; (L^+^) – With irradiation

**Reduction of exopolysaccharides**

| **Experiment Conditions** | ***E. coli*** | | | | ***S. aureus*** | | | |
| --- | --- | --- | --- | --- | --- | --- | --- | --- |
|  | 1 | 2 | 3 | Mean | 1 | 2 | 3 | Mean |
| **MSN (L^-^)** | 8.11 | 6.34 | 6.88 | **7.11 ± 0.90** | 7.25 | 7.98 | 6.48 | **7.24 ± 0.75** |
| **MSN (L^+^)** | 12.13 | 12.50 | 14.86 | **13.16 ± 1.47** | 12.86 | 11.05 | 13.63 | **12.51 ± 1.32** |
| **MG (L^-^)** | 16.02 | 15.74 | 14.20 | **15.32 ± 0.98** | 12.57 | 14.92 | 14.78 | **14.09 ± 1.31** |
| **MG (L^+^)** | 28.63 | 29.58 | 26.87 | **28.36 ± 1.37** | 24.08 | 21.89 | 26.69 | **24.22 ± 2.40** |
| **MG-MSN (L^-^)** | 20.76 | 20.98 | 17.86 | **19.87 ± 1.74** | 22.42 | 24.31 | 26.66 | **24.46 ± 2.12** |
| **MG-MSN (L^+^)** | 41.03 | 43.19 | 46.49 | **43.57 ± 2.75** | 51.67 | 52.36 | 56.78 | **53.60 ± 2.77** |

*Values are expressing the percentage of inhibition

*(L^-^) – Without irradiation; (L^+^) – With irradiation
